# Parry Romberg syndrome with a wide range of ocular manifestations: a case report

**DOI:** 10.1186/s12886-015-0093-0

**Published:** 2015-09-05

**Authors:** Antonio Maria Fea, Vittoria Aragno, Cristina Briamonte, Mauro Franzone, Davide Putignano, Federico Maria Grignolo

**Affiliations:** Department of Clinical Sciences, Ophthalmology Institute, University of Turin, Via Juvarra 19, 10100 Turin, Italy

## Abstract

**Background:**

Parry-Romberg syndrome (PRS) is a rare disorder characterized by unilateral facial atrophy affecting the skin, subcutaneous tissue, muscles, and sometimes extending to the osteocartilaginous structures. Ocular involvement is relatively rare.

**Case presentation:**

We present a case of a 23-year-old female caucasian patient with Parry Romberg syndrome and extensive ocular involvement: enophthalmos, uveitis, iris atrophy. Ultrasound biomicroscopy (UBM) demonstrated hypotrophy of the ciliary body. The ciliary body atrophy has been previously reported just once and can be an explanation for the hypotony, frequently present in these patients.

**Conclusions:**

Parry Romberg syndrome is a rare multidisciplinary disease. Our case presents a full spectrum of ocular manifestations. The pathogenesis of hypotonia is discussed.

## Background

Parry-Romberg syndrome (PRS) is a rare disorder characterized by unilateral facial atrophy affecting the skin, subcutaneous tissue, muscles, and sometimes extending to the osteocartilaginous structures [1–4]. Ophthalmic involvement is uncommon with the most frequent finding being enophthalmos [5–11].

We present a case of PRS with enophthalmos, uveitis, ciliary body hypotrophy and phacodonesis.

## Case presentation

**Fig. 1:**
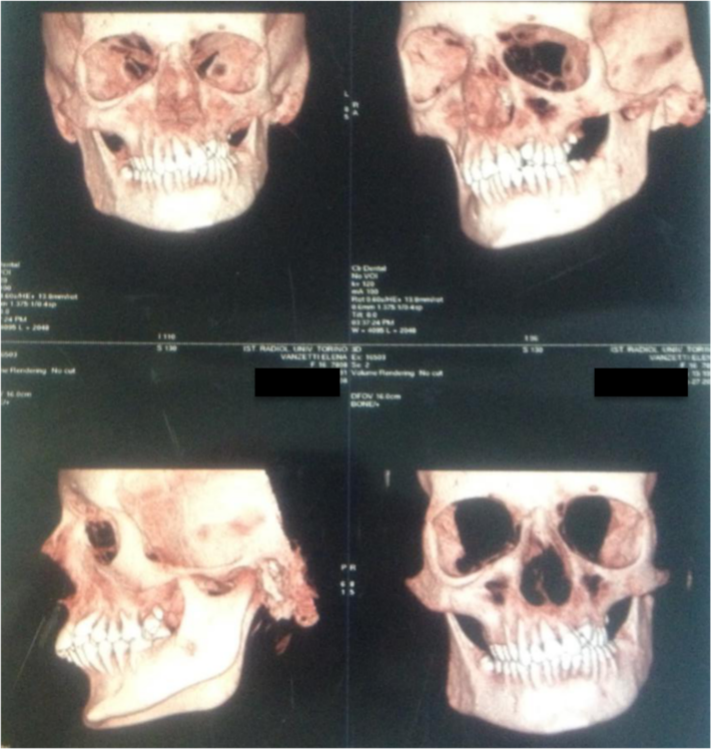
3D CT. 3D CT images show the normal anatomical right and deviated nasal septum, tilt in the occlusal table, hypoplastic zygomatic complex and orbital wall on left side

**Fig. 2:**
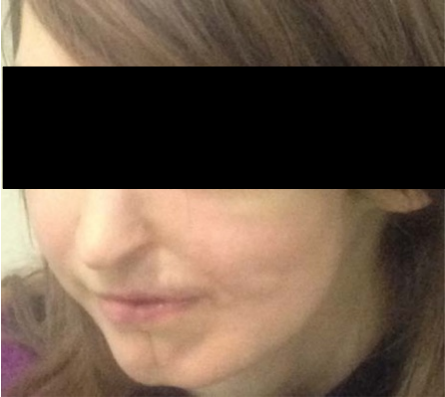
Patient image. Picture taken during our ophtalmic evaluation, showing the actual situation of the patient after two autologous fat injection

23 years old lady with 16 years history of Parry Romberg syndrome was referred to our clinic. The family history was positive for autoimmune diseases: her father was affected by rheumatoid arthritis and her first degree aunt by cutaneous psoriasis. No other cases of PRS were reported in the family. She did not report previous trauma, but had a Varicella Zoster virus (VZV) infection when she was 5 years old. When 6 years old, she developed a dermal hyperpigmented lesion in the left side of the neck. One year later she presented signs of left hemifacial atrophy. She progressively developed atrophy of cutaneous and subcutaneous fat tissue with maxillary-mandibular asimmetry and enophthalmos. The nasal septum and the mouth were deviated (Fig. 1). The disease progression stopped when she was 15 years old. She then underwent two autologous fat injections which reabsorbed after a few months. She didn’t undergo further surgical interventions. The disease is presently stable (Fig. 2). She complained migraine, but no other neurological symptoms. Serological tests were positive for anti-streptolysin O titre (369 U/ml; range 0-250 U/ml), rheumatoid factor (101 U/ml; range 0-40 U/ml), antibodies against nucleic (>1:640; range < 1:160) and against double strain DNA (42 U/ml; range < 30 - >50). The rheumatologic examination was negative. TSH was mildly elevated (4.27 UI/ml; range 0.2-4 UI/ml), but T3 and T4 were still in the normal range (T3:3.29 pg/ml; range 2.35-4.55 pg/ml and T4: 13.12 pg/ml; range 6.95-18.20 pg/ml), suggesting a subclinical hypothyroidism.

The ocular involvement started simultaneous to the development of facial atrophy. She first reported intense photofobia and she presented with multiple episodes of left iridocyclitis in the following years. She was diagnosed with Fuchs heterochromic iridocyclitis.

**Fig. 3:**
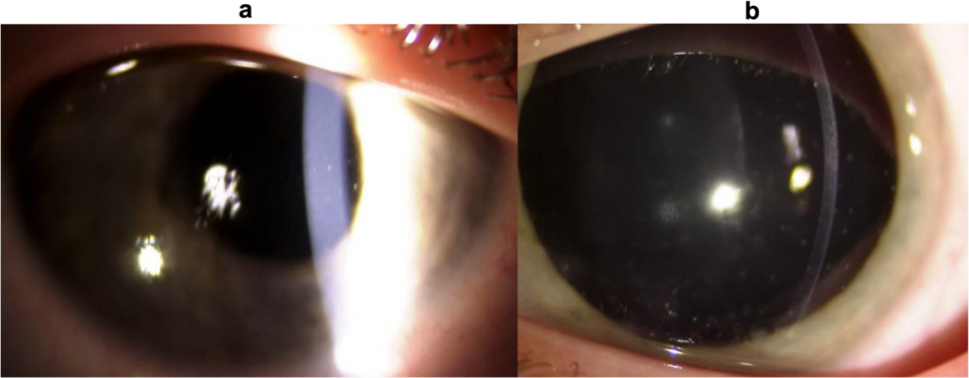
Slit lamp anterior segment photography. Anterior segment photos show bilateral non pigmented endothelial deposits (**a** right eye, **b** left eye); iris atrophy, partial aniridia and fully dilated pupil on left eye

**Fig. 4:**
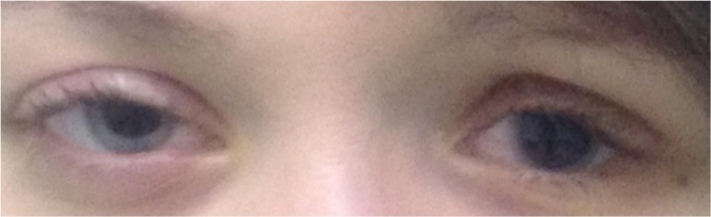
Aniridia. Picture showing differences between two eyes in terms of aniridia and pupil size

**Fig. 5:**
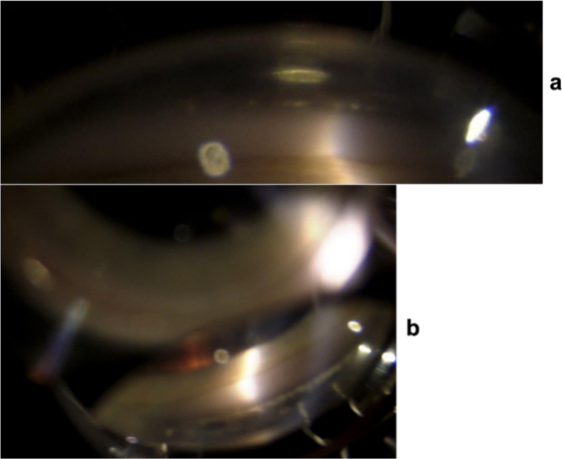
Gonioscopy. Gonioscopy images showing a closed angle in superior (**a**) and inferior (**b**) sectors

**Fig. 6:**
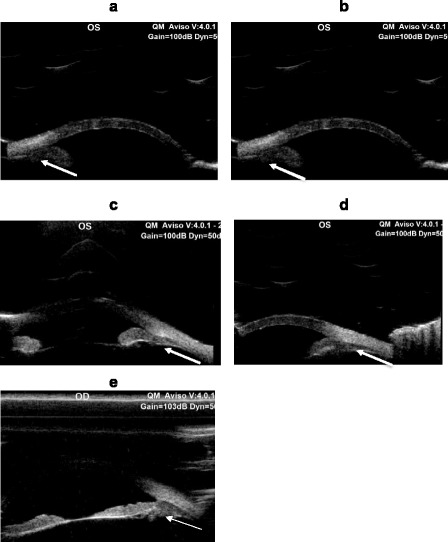
UBM images of left eyes taken at 3-6-9-12 o’clock respectively (**a**, **b**, **c**, **d**) showing a narrow angle at 3-6 and 12 o’clock and a closed angle at 9 o’clock. The ciliary body is hypotrophic (coarser white arrows). UBM image of right eye (8**e**) showing a dystrophic ciliary body (thinner white arrows)

She presented to our Clinic with left enophthalmos and pseudoptosis. Her best corrected visual acuity was 20/20 in the right eye and 20/60 in the left eye. The anterior segment examination showed bilateral non-pigmented endothelial deposits (Fig. 3a and b), but no flare. The left iris was atrophic with partial aniridia, the pupil was non reactive to light (Fig. 4). The anterior chamber depth was reduced in periphery. Gonioscopy showed a narrow angle grade 1‐2 (Shaffer) (Fig. 5a and b) and ultrasound biomicroscopy (UBM) demonstrated a narrow angle at 3,6 and 12 o’clock and a closed angle at 9 o’clock. Unexpectedly during this exam, the ciliary body was atrophic (Fig. 6a,b,c,d). The right eye UBM reported a dystrophic ciliary body (Fig. 6e).

The lens showed phacodonesis. The intraocular pressure (IOP) measured with Goldmann applanation tonometry was 12 mmHg in the right eye and 10 mmHg in the left eye. Fundus examination was normal in both eyes.

## Conclusions

Parry-Romberg syndrome (PRS) is a rare disorder characterized by atrophy of cutaneous, subcutaneous fatty tissue, muscle tissue and rarely bone structures. It is more common in women, it usually involves one side of the face, the ipsilateral involvement of the body is rare and 20 % of cases are bilateral [9]. PRS usually begins in the first decade of life [1–3, 12], although some cases with a late onset have been described [12], and is slowly progressive over two to twenty years and then stabilizes [1, 2, 13–16]. Our patient shows a typical onset in childhood and the progression was slow in the following ten years.

PRS etiology in still unknown: traumatic, genetic, immune mediated processes, hormonal disregulation, infection (Herpes and *Borrelia burgdorferi* mostly involved [17–19]) and sympathetic dysfunction have been proposed. In our case we found an history of familial genetic predisposition to develop autoimmune disease, a serological assessment demonstrated a potential immune and hormonal disregulation, and a positive history of Varicella Zoster Virus infection preceding the PRS.

As referred in literature, an early sign of PRS is a frontal hypo or hyperpigmented skin lesion known as “frontal linear scleroderma *en coupe de sabre*”, a localised form of sclerodermia. In our case the only sign preceding the developement of the atrophy was a hyperpigmented neck lesion. Frontal linear sclerodermia (LSCS) and PRS have the same starting age, disease progression, ocular and neurological involvement and predominantly affect women. The prevalence of LSCS in conjunction with PRS is uncertain, but has been reported to range from 36.6 % to 53.6 % [20]. For this reason it has been suggested that PRS is a form of scleroderma, although some Authors believe that they are separate entities [20–23].

PRS often coexists with neurological, cardiac, ophtalmological, rheumatological, endocrine, maxillo-facial and orthodontal disorders [7]. Neurological involvement is the most common association [24, 25] including migraine, hemiplegia, brain atrophy, and intracranial vascular anomalies. For this reasons some Authors consider the PRS as a neuro-cutaneous syndrome [24, 25]. In our case migraine was the only neurological symptom presented by the patient.

Several ophthalmologic associations have been previously described, affecting 10-30 % of patients, the most common being enophthalmos, eyelid atrophy, uveitis, retinal vasculitis, oculo-motor defects and glaucoma. Other findings are presented in Table 1.

**Table 1:** Ocular findings in Parry Romberg disease

Ocular structure	Ocular manifestation
Orbit	Enophtalmos [7–30]
	Orbital neurinoma [31]
Extraocular muscles	Third nerve paralysis (ipsi or controlateral) [13]
	Muscle thinning [32]
	Exo-eso-ipotropia [33–35]
	Diplopia [33]
	Restrictive strabismus [36]
Eyelids	Retraction [35]/lagophthalmos
	Atrophy [23], Pseudoptosis [37]
Cornea	Band keratopathy [30, 38]
	Exposure keratopathy [39]
	Primary endothelial failure [40]
	Ipoestesia [30]
	Endothelial precipitates [41]
	Flourlike stromal deposits [42]
Iris/Uvea	Uveitis [20]
	Fuchs heterochromic iridocyclitis [38]
	Iris atrophy (our case, [33, 43])
Lens	Cataract [38]
	Lens dislocation (our case, [26])
Ciliary body	Hypotony/phtisis [27]
	Atrophy (our case, [26])
	Glaucoma [28]
Vitreous	Bilateral vireitis [44]
Retina	Retinal vasculitis [45, 46]
	Retinal telangiectasia [32]
	Retinal pigment epithelial changes [34]
	Retinal detachment [47]
	Coats disease [44, 47]
	Chorioretinal lesions [48]
	Central retinal artery occlusion [49]
Optic nerve	Papillitis [44]/neuroretinitis [44]
Neuro-ophthalmic	Horner syndrome [21]
	Adie pupil [25]
	Mydriase/miosis [21]

Our patient had an extensive ophthalmologic involvement: enophthalmos, pseudoptosis, uveitis, iris atrophy and partial aniridia, narrow angle, phacodonesis and ciliary body hypotrophy. Phacodonesis has never been reported before and ciliary body atrophy has recently been reported by Ashwini Kini et al. (2015) [26].

The ciliary body hypotrophy should have led to ocular hypotony, but the IOP was similar in both eyes. The presence of the narrow angle and thus a reduction of outflow may compensate for the decrease of inflow. Alternatively, the ciliary body, although atrophic, might be still functional and its production of acqueous humor sufficient to prevent hypotonia.

Previous Authors reported occurrence of hypotony or glaucoma in patients with the Parry Romberg Syndrome. Hypotony is generally considered as consequent to an edema of the ciliary body due to uveitis [27]. The occurrence of glaucoma has been considered secondary to uveitis or angle closure [28].

Our findings of ciliary body hypotrophy was occasional. We were evaluating the chamber angle with UBM and we came across this novel finding.

UBM examination is in general seldom used in the ophthalmic practice and ciliary hypotrophy might be more frequent than expected in this Syndrome, which affects mesodermic and ectodermic tissues.

We suggest that UBM examination should be further used in anterior segment malformations associated with the Parry Romberg syndrome.

## Consent

Written informed consent was obtained from the patient for publication of this Case Report and any accompanying images. A copy of the written consent is available for review by the Editor of this Journal.

## Abbreviations

PRS: Parry-Romberg syndrome
LSCS: Frontal linear sclerodermia
IOP: Intraocular pressure
UBM: Ultrasound biomicroscopy
